# Equine West Nile encephalitis, United States.

**DOI:** 10.3201/eid0704.010412

**Published:** 2001

**Authors:** E N Ostlund, R L Crom, D D Pedersen, D J Johnson, W O Williams, B J Schmitt

**Affiliations:** Animal and Plant Health Inspection Service, U.S. Department of Agriculture, 1800 Dayton Ave., Ames, IA 50010-0844, USA. eileen.n.ostlund@aphis.usda.gov

## Abstract

After the 1999 outbreak of West Nile (WN) encephalitis in New York horses, a case definition was developed that specified the clinical signs, coupled with laboratory test results, required to classify cases of WN encephalitis in equines as either probable or confirmed. In 2000, 60 horses from seven states met the criteria for a confirmed case. The cumulative experience from clinical observations and diagnostic testing during the 1999 and 2000 outbreaks of WN encephalitis in horses will contribute to further refinement of diagnostic criteria.


### Equine West Nile Encephalitis, United States

**Eileen N. Ostlund,* Randall L. Crom,† Douglas D. Pedersen,* Donna J. Johnson,* W. Oliver Williams,† and Beverly J. Schmitt***

*Animal and Plant Health Inspection Service, U. S. Department of Agriculture, Ames, Iowa, USA; and †Animal and Plant Health Inspection Service, U. S. Department of Agriculture, Riverdale, Maryland, USA

After the 1999 outbreak of West Nile (WN) encephalitis in New York horses, a case definition was developed that specified the clinical signs, coupled with laboratory test results, required to classify cases of WN encephalitis in equines as either probable or confirmed. In 2000, 60 horses from seven states met the criteria for a confirmed case. The cumulative experience from clinical observations and diagnostic testing during the 1999 and 2000 outbreaks of WN encephalitis in horses will contribute to further refinement of diagnostic criteria.

In October 1999, West Nile (WN) virus was first confirmed as the cause of illness in a horse in the Western Hemisphere. A Suffolk County, New York, horse was 1 of 25 on Long Island that were eventually diagnosed with WN encephalitis in 1999. Nine (36%) of the infected horses died or were euthanized.

A limited number of veterinary diagnostic tests were available during the 1999 outbreak. Few laboratories were prepared to conduct diagnostic tests, in part because WN virus is categorized as a biosafety level 3 agent (1). The United States Department of Agriculture, Animal and Plant Health Inspection Service (APHIS), Veterinary Services, National Veterinary Services Laboratories (NVSL), Ames, Iowa, which is an international veterinary reference laboratory for diagnosis of eastern, western, and Venezuelan equine encephalomyelitis (EEE, WEE, VEE), provided diagnostic tests for WN virus. Plaque-reduction neutralization tests (PRNT) of equine serum (2) and virus isolation from equine brain or spinal cord tissues were the primary WN virus laboratory methods available in 1999. Most submissions to the NVSL in 1999 were coordinated by APHIS Veterinary Services as exotic disease investigations.

Evidence gathered during the winter of 1999-2000 indicated that WN virus was still present in birds and mosquitoes in the New York City area. In early February 2000, WN virus was isolated from a Red-tailed Hawk that died in Westchester County, New York (3). Adult *Culex* mosquitoes collected from structures in New York City during January and February 2000 were found to be infected with WN virus (4,5). Given these findings, epizootic levels of WN virus activity were thought likely to recur in the summer of 2000.

The laboratory methods used to detect WN virus infection and exposure in horses served well in the initial outbreak in 1999. However, with evidence that the virus had become established in the northeastern United States, the number and range of horses exposed to WN virus were expected to increase. To facilitate detection of new equine WN virus infections, an immunoglobulin (Ig) M-capture enzyme-linked immunosorbent assay (MAC-ELISA) was developed. The assay, modeled after an EEE MAC-ELISA, used an inactivated WN virus antigen from neonatal mouse brain (6). Serum samples collected during the 1999 WN virus outbreak were used to validate the assay. Results of experimental challenge of a small number of horses showed that IgM isotype anti-WN virus antibodies became detectable 8-10 days after infection and persisted <2 months in the challenge model (Ostlund et al., unpub. data). Based on sequential samples collected from a few horses in the New York WN virus outbreak in 1999, the decay of IgM antibodies in naturally infected horses appeared to be similar (7).

Given the possibility of equine cases of WN encephalitis recurring, a case definition was developed by APHIS Veterinary Services in the spring of 2000. Clinical signs used in the definition were based primarily on the 1999 experience in the United States because descriptions of clinical equine cases of WN encephalitis in other parts of the world were limited (8-12). To assure comparability and consistency of results, all diagnostic testing referred to in the case definition was required to be performed or confirmed in the same laboratory. Because specimens might originate from multiple states, the laboratory designated to test all specimens was the NVSL.

We evaluated diagnostic test results in combination with clinical observations to accurately identify cases of WN encephalitis in horses.

### Methods

### Characterization of Clinical Illness

Dates of onset and signs of clinical illness were obtained from field investigators' interviews with animal owners or care givers or from history forms accompanying specimens submitted to primary or reference diagnostic laboratories. Date of onset of illness was considered to be the first time at which any sign of illness was observed that led to a sign specified in the case definition.

### Laboratory Tests

Specimens included serum, whole blood, cerebrospinal fluid (CSF), brain, and spinal cord tissue. Not all submissions included each sample type.

Address for correspondence: Eileen N. Ostlund, National Veterinary Services Laboratories, P.O. Box 844, 1800 Dayton Ave., Ames, Iowa 50010-0844, USA; fax: 515-663-7348; e-mail: eileen.n.ostlund @aphis.usda.gov

Virus isolation in rabbit kidney and Vero cell cultures was attempted from brain and spinal cord tissue samples, as well as from whole blood (13). Two cell culture passages were performed with each cell line, and cultures were examined daily for cytopathic effect. WN virus isolates were confirmed by fluorescent antibody testing of infected cell cultures with a monoclonal antibody. In addition, virus isolation attempts for some submissions included intracerebral inoculation of 8 to 16 neonatal mice. When examination for other equine pathogens was indicated (e.g., virus isolates not identified as WN virus), additional virologic tests to identify equine herpesvirus-1 (EHV-1), EEE, WEE, or VEE were performed. EHV-1 isolates were confirmed by fluorescent antibody (14), and alphavirus isolates were identified by complement fixation tests (15).

The 1999 New York avian and equine WN virus isolates were cytopathic in cell culture and formed plaques when cultures were overlaid with agar; a crow isolate was selected as the NY99 prototype for the PRNT. Serum dilutions of 1:10 and 1:100 were examined for WN virus neutralizing antibodies by PRNT in 25-cm$^2$ flasks (2). One hundred PFU of WN virus were used in the test. Briefly, virus-serum mixtures were incubated at 37°C for 75 minutes and then added to flasks containing confluent monolayers of Vero cells. Following incubation at 37°C for 60 minutes, flasks were overlaid with agar and incubated an additional 72 hours. A second agar overlay containing neutral red was then added, and the flasks were examined the following day. Plaque reduction $\geq$90% was recorded as positive. A PRNT titer at least 1:10 in equine serum was considered significant (16).

WN virus-specific IgM antibodies in CSF and sera were measured by MAC-ELISA (6,13). All reagents were titrated for optimal performance in the assay. Sera were tested in duplicate at dilutions of 1:100 and 1:1,000; CSF was tested at dilutions of 1:2 and 1:20 in the MAC-ELISA. Briefly, microtiter plates (Immulon 1B, Dynex, Chantilly, VA) were coated with anti-equine IgM (Kierkegaard & Perry Laboratories, Gaithersburg, MD) and blocked with 5% nonfat dry milk. Serum and CSF samples were allowed to bind to the capture antibody overnight at 4°C. After washing, bound equine IgM was reacted with WN virus antigen and control antigen prepared from infected and normal neonatal mouse brain, respectively. After incubation and washing, a flavivirus-specific horseradish peroxidase antibody conjugate (Centers for Disease Control and Prevention, Atlanta, GA) was added. Bound conjugate was detected by reaction with 2,2'-azino-bis(3-ethylbenzthiazoline-6-sulfonic acid) as the enzyme substrate. The absorbance at 410 nm was measured, and antigen-specific reactions exceeding twice the negative control were considered positive.

### Criteria for Confirmed and Probable Equine WN Encephalitis Cases

According to the APHIS Veterinary Services WN encephalitis case definition, a confirmed case was illness in an equine with clinical signs plus one or more of the following: isolation of WN virus from tissue, blood, or CSF; an associated fourfold or greater change in PRNT antibody titer to WN virus in appropriately timed, paired sera; or detection of both IgM antibody to WN virus by MAC-ELISA and an elevated titer (positive at $\geq$1:10) to WN virus antibody by PRNT in a single serum sample.

A probable case was an equine with clinical signs, located in a county in which WN virus has been confirmed in the current calendar year in any population: mosquito, bird, human, or horse (or within 10 miles of a current-year confirmed equine case), plus one or more of the following: detection of IgM antibody to WN virus by MAC-ELISA but no elevated titer (negative at 1:10) to WN virus antibody by PRNT in a single serum sample taken $\leq$21 days after onset of illness; positive polymerase chain reaction (PCR) for WN virus genomic sequences in tissue, blood, or CSF; or positive immunohistochemistry for WN virus antigen in tissue. Clinical signs must include one or more of the following: ataxia (including stumbling, staggering, wobbly gait, or incoordination), inability to stand, multiple limb paralysis, or death.

### Results

From January 2000 through January 2001, samples from approximately 360 horses for which viral encephalitis was among the differential diagnoses were submitted to the NVSL. Submissions originated from federal, state, university, and private diagnostic laboratories and veterinary practitioners. Eighty-eight submissions contained equine brain or spinal cord tissue; 314 contained serum, CSF, or both, with some submissions including samples for both virologic and serologic tests. The submissions originated from 33 states, with most from the northeastern United States.

In 2000, 60 horses were classified as having a confirmed case of WN encephalitis. Twenty-three (38%) of the 60 cases were fatal, as the horses either died or were euthanized. Clinically, both central nervous system (CNS) and peripheral nervous system (PNS) signs were reported (Table). Most ill horses were reported to be ataxic. Two other common signs included weakness of limbs or going down with difficulty rising. Signs more commonly reported in 2000 than in 1999 included muscle fasciculation, fever, facial paralysis, facial twitching, teeth grinding, and blindness.

Brain tissue samples were submitted from 10 of 60 horses, and CSF was submitted from 6. WN virus was isolated from brain tissue of seven horses that became ill in August or September 2000. Although not included among the diagnostic tests for WN virus case confirmation in 2000, all brain samples were also tested for WN virus RNA by reverse transcription (RT)-nested PCR (RT-nPCR) (17). Brain samples from each of the seven horses yielding a WN virus isolate, plus an additional three confirmed equine WN virus cases, were RT-nPCR positive for WN virus RNA.

No brain samples yielded more than one viral pathogen. However, concurrent with WN virus isolations, EEE virus

Table. Clinical signs in horses with West Nile encephalitis, 2000

| Clinical sign | Percentage of horses with sign |
|---|---|
| Ataxia | 85 |
| Weakness of limbs | 48 |
| Recumbency, difficulty rising, or both | 45 |
| Muscle fasciculation | 40 |
| Fever | 23 |
| Paralyzed or drooping lip | 18 |
| Twitching face or muzzle | 13 |
| Teeth grinding | 7 |
| Blindness | 5 |

was isolated from 16 equine brain samples submitted in the fall of 2000. The EEE-positive samples were from New Jersey, North Carolina, South Carolina, and Virginia. EHV-1 was isolated in June from the plasma of one horse from Vermont with neurologic illness. WN virus serologic tests were uniformly negative in horses from which EEE or EHV-1 was isolated.

Serum was submitted for all 60 confirmed equine WN encephalitis cases identified in 2000. Fifty-nine of 60 horses had demonstrable WN virus-specific IgM antibodies in acute-phase serum samples. All six CSF samples had detectable IgM antibodies to WN virus when tested at a dilution of 1:2, and three of six CSF samples tested WN virus IgM-positive at the 1:20 dilution.

Neutralizing antibody titers $\geq$1:10 were detected in 55 of 59 serum samples collected at initial visit; insufficient serum was received from one horse to test at the 1:10 dilution by PRNT. Acute-phase serum samples from two of four horses that did not have detectable neutralizing antibody did have IgM antibody to WN virus. Both these horses had fatal cases of encephalitis, and WN virus was isolated from brain tissue.

The illnesses of two horses originally classified as probable cases were later reclassified as confirmed based on additional test results. The first horse was IgM positive ($\geq$1:1,000) but PRNT negative on a serum sample taken 2 days after clinical onset; a second serum sample drawn 14 days later had a PRNT titer of $\geq$1:100. The second horse was IgM positive ($\geq$1:1,000), but PRNT negative at 1:100 with insufficient serum drawn on the first day of clinical illness to test at other dilutions; a second serum sample drawn 22 days later had a PRNT titer of 1:10. One horse met the confirmed case definition by a greater than fourfold change in PRNT titers in paired samples. The PRNT titer in the acute-phase serum was 1:10, and a subsequent sample had a PRNT titer of $\geq$1:100.

Cases of WN encephalitis identified in 2000 had onset of illness from mid-August to the end of October, with 42 (70%) of the 60 cases occurring in a 4-week period from mid-September to mid-October (Figure). Cases were detected in seven northeastern states, six of which had no equine cases of WN encephalitis identified in 1999. Forty-six (77%) of the cases were in New Jersey or New York. Horses ranged in age from 4 months to 38 years (mean 14.0 years). Thirty-six of the horses were male (32 geldings, 3 stallions, 1 colt), and 24 were mares. Cases occurred in at least 11 breeds of horses.

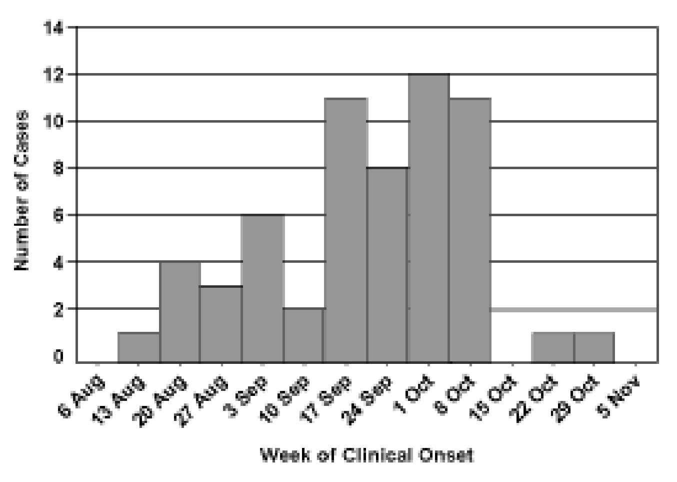

Figure. Equine cases (n=60) of West Nile encephalitis in the United States, by week of clinical onset, 2000.

### Conclusion

The emergent nature of WN virus in the United States necessitates reevaluation of the case definition to accommodate clinical observations and additional laboratory methods. Similar to events in human medicine over the past 2 years, valuable diagnostic experience has been acquired to facilitate identification of WN encephalitis in the U.S. equine population. Advances in recognition of clinical signs associated with WN encephalitis in horses and new laboratory diagnostic tests continue to contribute to improved veterinary diagnostic capability.

In 1999, virus isolation and neutralizing antibody detection tests were used to test suspected cases of equine WN encephalitis. In 1999, WN virus was isolated from brain or spinal cord from three horses with WN encephalitis (18). Virus isolates were identified by reverse transcription PCR with RNA extracted from infected cell cultures (19), confirming WN virus as the flavivirus responsible for the 1999 equine epizootic.

In surveillance and diagnostic testing of horses possibly exposed to WN virus in 1999, no serum samples tested from equine submissions from New York counties outside the outbreak area or from 22 other states contained detectable neutralizing WN virus antibodies. To date, there is no evidence that WN virus occurred in the U.S. equine population before 1999.

Neutralizing antibodies to WN virus may persist for $\geq$2 years following infections in humans (8). In limited samples collected in New York from horses that were seropositive in 1999, neutralizing antibody was commonly detected through the following winter (7). More recent testing of the same horses indicates that their WN virus neutralizing antibodies have now persisted for at least 15 months (Ostlund et al., unpub data). Such enduring titers, while perhaps engendering protection from reinfection, have the potential to complicate serologic diagnosis of new infections in a geographic area where WN virus activity had previously occurred. Since all WN virus infections in equines may not give rise to clinical disease, the serologic status of inapparently infected horses is likely to be unknown. Development of subsequent neurologic disease in such an animal could be mistaken for WN virus infection based on persistent neutralizing antibody in the serum. Transfer of WN virus-neutralizing antibodies via colostrum from a seropositive mare in New York to her foal was demonstrated in the spring of 2000 (authors' unpub. observations). Taken together, these data indicate that detection of WN virus-specific neutralizing antibody in a single equine serum sample has limited diagnostic value for new infections in regions where WN virus infections have occurred in previous years.

To assist in identification of recent WN virus infections, the MAC-ELISA method was developed and incorporated into the repertoire of laboratory tests conducted on equine serum and CSF in 2000. Although the kinetics of equine IgM antibody responses to natural WN virus infection were largely unknown, IgM serum antibody responses were expected to wane more rapidly than neutralizing responses to WN virus.

Confidence in confirming equine WN encephalitis cases in the United States was enhanced by the concordance of multiple laboratory test results, including virus isolation. The number of WN virus isolates from horses in North America since 1999 has exceeded all previously published reports of the disease in horses worldwide. Nearly all infections of WN virus in horses in 2000 were confirmed by at least two laboratory test methods, with the combination of MAC-ELISA and PRNT serologic tests the most dependable in confirming cases in living horses. Fifty-four of 60 confirmed cases had detectable WN virus IgM and neutralizing antibody responses in the acute-phase serum samples. For submissions yielding WN virus from brain, positive WN virus RT-nPCR, or both, IgM was consistently present in serum. Eight of 10 submissions that were WN virus isolation positive, RT-nPCR positive, or both had neutralizing antibody in serum at the time of death. Convalescent-phase serums were needed to support confirmation in three cases. When available, CSF samples from acute-phase cases also yielded positive WN virus IgM results, although testing at a lower dilution than for serum was necessary.

During the 1999 equine outbreak, the primary clinical sign reported (in 18 of 25 cases) was ataxia, either sudden or progressive. Fever associated with clinical illness was documented in only one horse. There were attitudinal changes in many horses, including somnolence, listlessness, apprehension, depression, or periods of hyperexcitability. A greater range of clinical signs were reported for infected horses in 2000 than in the 1999 epizootic.

For a few WN virus suspected cases, the criteria for classification as a confirmed case were only partially met. Illnesses in horses that had only clinical signs not included in the 2000 case definition were not classified as cases by APHIS Veterinary Services. Three clinically ill horses had serum specimens with WN virus IgM titers $\geq$1:100 and PRNT titers $\geq$1:10, but none of the signs reported were compatible with the APHIS case definition. Although WN virus infection likely did occur in these horses, they were not included in the WN virus equine encephalitis cases for 2000, since their illness did not reflect encephalitis. In addition, some suspected WN virus cases could not be confirmed in the laboratory because of insufficient samples.

For some horses, the clinical history and geographic location prompted consideration of WN encephalitis but no laboratory tests supported such a diagnosis. Multiple cases of EEE were identified at the National Veterinary Services Laboratory in the fall of 2000, concurrent with the WN virus epizootic. Thus, considering other causes of equine neurologic disease in the differential diagnosis is important.

The surveillance case definition for WN encephalitis in equines used by APHIS Veterinary Services was developed to be as sensitive as possible, yet minimize false-positive case classifications. One of the primary reasons for performing surveillance for equine WN encephalitis is to be able to meet international obligations for disease reporting. Such disease reports can have substantial ramifications for the international movement of horses and other livestock. A high level of specificity in case classification is therefore critical, especially when detecting and reporting the first case of disease in a given geographic area (e.g., a previously unaffected state). Given the specificity of the case definition, failure of a clinically ill equine to meet the criteria for a probable or confirmed case does not completely exclude the possibility that WN virus was the cause of illness.

Based on experience gained in 2000, some modifications will be considered in the diagnostic tests and clinical observations used to identify cases of WN encephalitis in horses in 2001 and future years. Such changes include the addition of a wider range of clinical signs, including PNS signs and additional CNS signs. New laboratory tests will also be incorporated, in particular RT-nPCR, which has been shown to be accurate in detecting WN virus nucleic acid in CNS tissues.

As the range of WN virus activity increases, prevention and control issues for horses becomes even more important. To date, prevention recommendations have been broad and generally targeted at reducing sources of water for mosquito breeding and decreasing equine exposure to biting mosquitoes. Risk factors for equine infection with WN virus are being evaluated through a case-control study conducted by APHIS and animal health officials in states where equine WN encephalitis was detected in 2000. Results of that study may provide information for more specific recommendations on preventing equine infections. Of most use in preventing illness and death of equines may be a vaccine against WN virus. Vaccines for equine use are being developed and could be available for use as early as the summer of 2001.

### Acknowledgments

The authors thank Kevin Lake and Kathryn Moser for superb technical support in equine sample testing and Robert E. Shope for providing monoclonal antibody. The authors also thank the numerous field investigators and persons who submitted diagnostic samples, particularly John E. Andresen.

Dr. Ostlund heads the Equine and Ovine Viruses Section, Diagnostic Virology Laboratory, National Veterinary Services Laboratory, where she coordinates West Nile virus diagnostic activities. She serves as an Office International des Epizooties designated expert on eastern, western, and Venezuelan equine encephalomyelitis.

### References

1. Richmond JY, McKinney RW, editors. Biosafety in microbiological and biomedical laboratories. 4th ed. US Department of Health and Human Services (US). Washington: US Government Printing Office; 1999.
2. Beaty BJ, Calisher CH, Shope RE. Arboviruses. In: Schmidt NH, Emmons RW, editors. Diagnostic procedures for viral, rickettsial and chlamydial infections. 6th ed. Washington: American Public Health Association, Inc.; 1989. p. 797-856.
3. Garmendia AE, Van Kruiningen HJ, French RA, Anderson JF, Andreadis TG, Kumar A, et al. Recovery and identification of West Nile virus from a hawk in winter. J Clin Microbiol 2000;38:3110-1.
4. Centers for Disease Control and Prevention. Update: surveillance for West Nile virus in overwintering mosquitoes—New York, 2000. MMWR Morb Mortal Wkly Rep 2000;49:178-9.
5. Centers for Disease Control and Prevention. Notice to readers: update: West Nile virus isolated from mosquitoes—New York, 2000. MMWR Morb Mortal Wkly Rep 2000;49:211.
6. Sahu SP, Alstad AD, Pedersen DD, Pearson JE. Diagnosis of eastern equine encephalomyelitis virus infection in horses by immunoglobulin M and G capture enzyme-linked immunosorbent assay. J Vet Diagn Invest 1994;6:34-8.
7. Ostlund EN, Andresen JE, Andresen M. West Nile encephalitis. Vet Clin North Am Equine Pract 2000;16:427-41.
8. Hayes CG. West Nile fever. In: Monath T, editor. The arboviruses: epidemiology and ecology. Vol. V. Boca Raton (FL): CRC Press; 1989. p.59-88.
9. Pan American Health Organization. West Nile fever. In: Acha PN, Szyfres B, editors. Zoonoses and communicable diseases common to man and animals. 2nd ed. Washington: Pan American Health Organization; 1987. p. 525-8. Scientific pub. no. 503.
10. Hubálek Z, Halouzka J. West Nile fever—a reemerging mosquito-borne viral disease in Europe. Emerg Infect Dis 1999;5:643-50.

11. Cantile C, Di Guardo G, Eleni C, Arispici M. Clinical and neuropathological features of West Nile virus equine encephalomyelitis in Italy. Equine Vet J 2000;32:31-5.
12. Schmidt JR, El Mansoury HK. Natural and experimental infection of Egyptian equines with West Nile virus. Ann Trop Med Parasitol 1963;57:415-27.
13. Pearson JE. Equine encephalomyelitis (Eastern and Western). In: Office International des Epizooties, Manual of standards for diagnostic tests and vaccines. 3rd ed. Paris: OIE Press; 1996. p. 400-5.
14. Yeargan MR, Allen GP, Bryans JT. Rapid subtyping of equine herpesvirus 1 with monoclonal antibodies. J Clin Microbiol 1985;21:694-7.
15. Department of Health, Education, and Welfare (US) Public Health Service. A guide to the performance of standardized diagnostic complement fixation method and adaption to micro test. Atlanta: Center for Disease Control; 1974.
16. Centers for Disease Control and Prevention. National West Nile Virus Surveillance System, 2000: final plan. May 26, 2000. Available from URL: http://www.cdc.gov/ncidod/dvbid/westnile/publications.htm
17. Johnson DJ, Ostlund EN, Pedersen DD, Schmitt BJ. Detection of North American West Nile virus in animal tissue by a reverse transcription-nested polymerase chain reaction assay. Emerg Infect Dis 2001;7:739-41.
18. Torres A. West Nile fever in the United States of America. Office International des Epizooties Disease Information 2000;13:5-7.
19. Lanciotti RS, Kerst AJ, Nasci RS, Godsey MS, Mitchell CJ, Savage HM, et al. Rapid detection of West Nile virus from human clinical specimens, field-collected mosquitoes, and avian samples by TaqMan reverse transcriptase-PCR assay. J Clin Microbiol 2000;38:4066-71.
